# Therapeutic Perspectives of Aminoflavonoids—A Review

**DOI:** 10.3390/ijms26052014

**Published:** 2025-02-25

**Authors:** Monika Stompor-Gorący, Agata Bajek-Bil, Natalia Potocka, Izabela Zawlik

**Affiliations:** 1 Department of Pathophysiology, Faculty of Medicine, Collegium Medicum, University of Rzeszów, Warzywna 1a, 35-310 Rzeszów, Poland; 2 Faculty of Chemistry, Rzeszów University of Technology, 35-959 Rzeszów, Poland; abajek@prz.edu.pl; 3 Laboratory of Molecular Biology, Centre for Innovative Research in Medical and Natural Sciences, Collegium Medicum, University of Rzeszów, 35-959 Rzeszów, Poland; npotocka@ur.edu.pl (N.P.); izawlik@ur.edu.pl (I.Z.); 4 Department of General Genetics, Faculty of Medicine, Collegium Medicum, University of Rzeszów, 35-959 Rzeszów, Poland

**Keywords:** aminoflavonoids, aminochalcone, aminoflavone, anticancer activity, antioxidants

## Abstract

Natural compounds containing nitrogen are a source of many biologically active molecules used as drugs. Due to their multidirectional effects, they represent effective therapeutic compounds in many medical areas. Flavonoids, as well as their bioprecursors, chalcones, that occur in plants possess a number of medicinal benefits. Their synthetic amino derivatives constitute a large group of compounds that exhibit pharmacological activity. Due to the increasing level of drug resistance among patients, new therapeutic agents and options are urgently needed. Therefore, aminoflavonoids may be a promising source of new drugs. In this review, the biological activities of flavonoids, including chalcones, with complexes containing a nitrogen atom and the aminoflavones Ru and Pt are summarized. The purpose of this review is to provide an overview of the synthesis and pharmacological activity of aminoflavonoids and to show how synthetic modifications of these compounds can influence their biological activities. It covers the most recent reports on obtaining aminoflavones, aminochalcones, and their derivatives, along with information about their anticancer, antimicrobial, antimalarial, antiviral, and anti-inflammatory activities.

## 1. Introduction

**Scheme 1:**
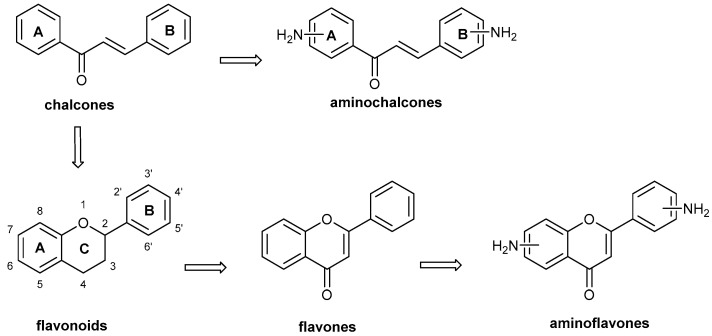
The general structures of flavonoids.

Flavonoids are biosynthesized in plants from malonate and the aromatic amino acids phenylalanine and tyrosine [1]. The basic flavonoid structure is the flavan nucleus, which consists of 15 carbon atoms arranged in three rings (C_6_-C_3_-C_6_), labeled A, B, and C (Scheme 1).

Various classes of flavonoids differ in the degree of oxidation and the pattern of substitution of the C-ring, while individual compounds in each class differ in the pattern of substitution of the A and B-rings. The flavonoid classes include flavones, flavanones, isoflavones, flavonols, flavanonols, flavan-3-ols, anthocyanidins, biflavones, chalcones, and aurones. In nature, most flavonoids are found as glycosides. The sugar moiety is a major determinant of the absorption of dietary flavonoid glycosides in humans [2].

Both synthetic and natural derivatives of flavonoids form an interesting and important group of molecules, as they possess a wide range of biological activities, such as anticancer, antioxidant, antibacterial, antiplasmodial, antiviral, antimalarial, anti-inflammatory, and many other actions [3,4,5]. Flavonoid compounds have also been reported to possess insect antifeedant, antiparasitic, antileishmanial, antifungal, and enzyme-inhibitory activities [6]. Additionally, chalcones and dihydrochalcones, being the precursors of natural flavonoids, can be used as potential cancer chemotherapy agents and inhibitors of enzymes involved in the metabolism of thyroid hormones [7,8].

The pharmacophoric substructures and physicochemical properties of the active compounds based on flavonoids have been defined—among them there are aromatic rings, a basic nitrogen atom, and high lipophilicity. Currently, many alkaloids containing a nitrogen atom are used as drugs. It is predicted that the antitumor effectiveness of a drug increases with the number of hydrogen bonds it forms with the cell membrane components, and hence a large number of known drugs contain nitrogen. There is also research on the functionalization of natural antioxidants, such as xanthohumol, for the synthesis of conjugates with biotin that contain nitrogen and sulfur atoms that are intended for use as anticancer therapeutics [9]. The nitrogen atom may influence the activity of the molecule through its contribution to the hydrogen bond strength [10]. Data show that the nitrogen atom plays an important role in the modulation of the multidrug resistance of cancer cells and in antimicrobial activity [11].

Many investigations [7,8,9,10] have shown that the introduction of aliphatic amino groups into the flavonoid moiety resulted in a potent antitumor activity, which is due to the enhancement of the aqueous solubility and drug-like character of candidate compounds. Much attention has been paid to flavonoids, but in the literature, we have not found any comprehensive review on aminoflavonoids. The aim of this review is to summarize the research on the preparation methods and biological activity of aminoflavones, aminochalcones, and their derivatives.

**Scheme 2:**
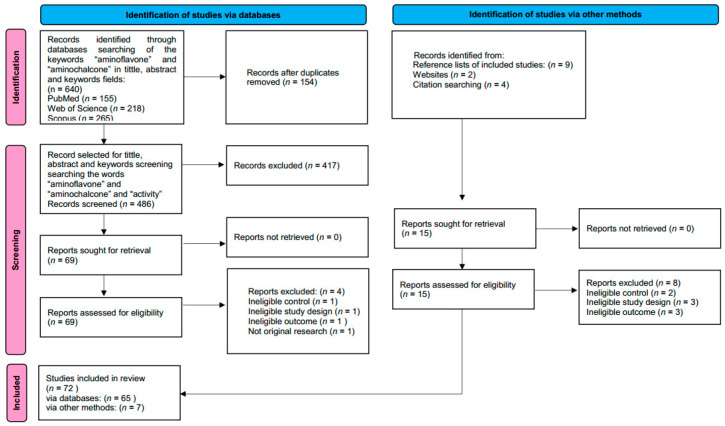
PRISMA flow diagram of inclusion of the review.

Selected scientific electronic databases, including PubMed, Scopus, and Web of Science, were used for searching the keywords, alone or in combination with other keywords. The flow diagram of the study selection process following the PRISMA guidelines is presented in Scheme 2.

## 2. Preparation of Aminoflavone Derivatives

Aminoflavones and aminoflavanones are a class of flavonoids that contain amino groups attached to the flavone or flavanone nucleus. They do not occur naturally and therefore must be synthesized via chemical methods [12,13].

**Scheme 3:**
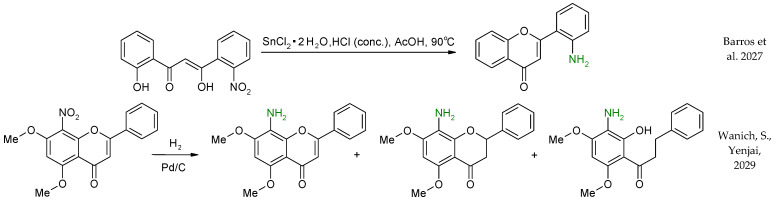

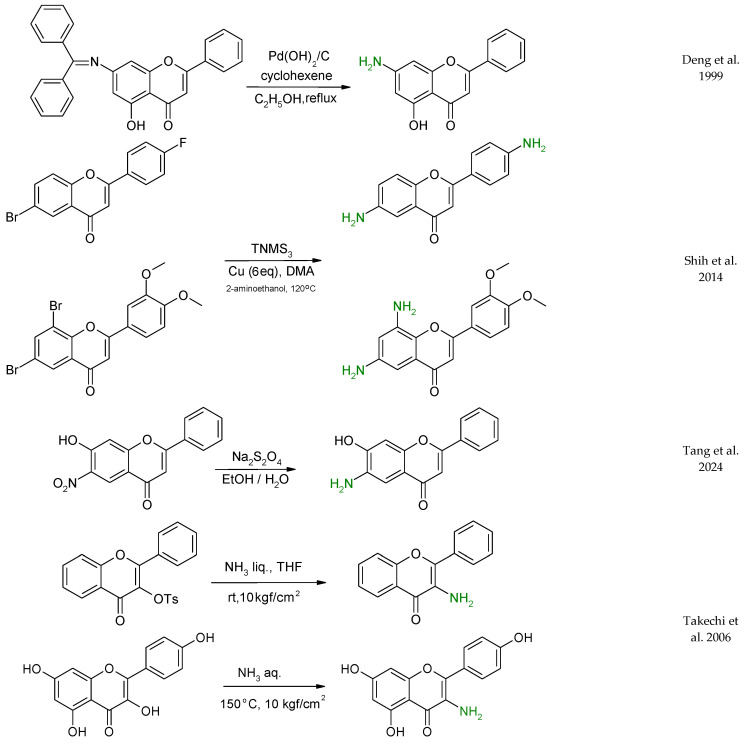
Selected chemical syntheses of aminoflavones [13,14,20,21,22,24].

A known method for the preparation of biologically active polyphenols with amino groups is the structural modification of natural compounds isolated from plants [14]. Nevertheless, reports on the synthesis and biological properties of aminoflavones are scarce [15,16,17]. These compounds can be obtained via the reduction of the corresponding nitro derivatives [18], by means of the total synthesis of the aminoflavone derivatives [12,19], by the reduction following the palladium-catalyzed cross-coupling reaction of the corresponding flavone triflate [20], via the reduction of nitroflavones [21], and from hydroxyflavones via tosyloxy- or mesyloxy flavones [22] (Scheme 3). Amino methylated derivatives of flavonoids can also be synthesized via the Mannich reaction upon the electrophilic substitution at C-8 with formaldehyde in the presence of either primary or secondary amines, in 2-propanol as the solvent [23]. Some of the compounds obtained in this way are promising inhibitors of α-glucosidase. An effective copper-mediated amination of bromoflavonoids, leading to a series of corresponding new aminoflavonoids, was also reported [24].

Researchers have made attempts to synthesize aminoflavonoids in order to make them available in high amounts or to add some functional groups that would enhance their biological or pharmacological effects [25]. Also, the biotransformation of these compounds is also the current area of interest, aiming to obtain novel biologically and pharmacologically active compounds based on aminoflavones [26], which contain a nitrogen atom in their structure.

## 3. Biological Activities of Aminoflavones

**Figure 1:**
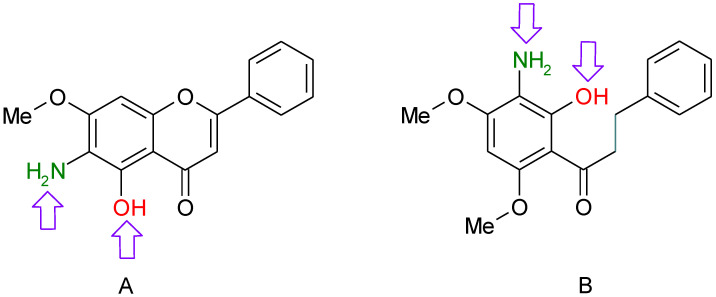
Chemical structure of (**A**) 6-amino-5-hydroxy-7-methoxyflavone and (**B**) 3′-amino-2′-hydroxy-4′6′-dimethoxy-α,β-dihydrochalcone.

According to the available literature, the compounds containing the aminoflavone moiety have good potential as anticancer agents. It is suggested that their cytotoxicity is due to the presence of amino and hydroxyl groups located at the ortho position (Figure 1) [14].

Also, aminochalcones, which are the precursors of aminoflavones, with a hydrophobic substituent in ring B proved to be potent antiproliferative agents [27].

One of the important objectives of medicinal chemistry is to design, synthesize, and produce molecules that have potential as human therapeutic agents. The synthesis of flavonoid derivatives containing a nitrogen atom is also an area of significant interest and many synthetic routes have been used to add various moieties to the natural flavonoid nucleus.

In the search for new agents to combat cancer, many compounds with the aminoflavone moiety have been synthesized and tested. Luzzani et al. [28] described the aminoflavone AFP 464, a new anticancer agent, which is currently undergoing phase II clinical trials as an antiproliferative compound active against human renal cancer cells at the molecular level (Section 3).

This compound also modulated the immune response in a mouse model study [29]. Brinkman et al. [30] showed that MDA-MB-468 and Cal51, both Erα-negative human breast cancer cell lines, are sensitive to growth inhibition mediated by another derivative of aminoflavone, which contains two amine groups in the A and B rings (e.g., 5-amino-2-(4-amino-3-fluorophenyl)-6,8-difluoro-7-methylchromen-4-one).

There is also some evidence that combining aminoflavones with other agents can enhance the efficacy of cancer treatment. Reinicke et al. [31] observed the synergistic activity of combinations of fluorinated diaminoflavone (5-amino-2,3-fluorophenyl)-6,8-difluoro-7-methyl-4H-1-benzopyran-4-one) and paclitaxel and some derivatives of topoisomerase I inhibitors (CAMP and SN38—metabolites of irinotecan) against MCF-7 human breast cancer cells.

**Figure 2:**
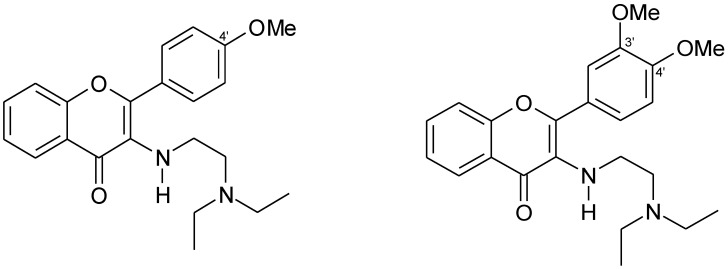
Examples of aminoflavones tested for their anticancer activity [33].

Dauzonne et al. [32] tested a series of aminoflavones for their cytotoxic activity. 3-Aminoflavones showed in vitro antiproliferative activity against murine L1210 leukemia cells with IC_50_ in the range of 10–163 µM. Among the tested 3-amino derivatives, the most potent were those bearing substituents in the 3′ and/or 4′-positions (Figure 2). The addition of the 3′-NH_2_ along with 4′-OCH_3_ groups doubled the activity of 3,3′-diamino-4′-methoxyflavone (IC_50_ = 10 µM) compared to the compounds with only the 4′-OCH_3_ substituent (3-amino-4′-methoxtflavone: IC_50_ = 22 µM). The presence of more than one methoxy substituent in the B phenyl ring resulted in a decrease in the activity. Substitution with the amino group at the 6-position in ring A (3,6-diaminoflavone) did not improve the anticancer potency.

The compounds where the 3-amino group was substituted with a dialkylamino group demonstrated similar activity to the corresponding 3-amino compounds.

Biological screening of amino-substituted flavone derivatives by Akama et al. [33] showed that 5,4′-diaminoflavone (Table 1) and some of its derivatives exhibited antiproliferative effects against the human breast cancer cell line MCF-7, irrespective of the presence or absence of estradiol (IC_50_ 0.0072 and 0.0098 µM, respectively).

Chemical modifications of 5-aminoflavone at the C-4′ position of ring B with more electron-withdrawing groups, such as cyano (-CN) and carboxy (-COOH) ones, resulted in a decrease in the activity. Similarly, acetylation of the 4′-amino group or relocation of the 4′-amino group to the 3′-position resulted in a decrease in the activity. Mono- and dialkylation of the 4′-amino group enhanced the antitumor activity; however, alkylation of this group is not essential for exhibiting its antiproliferative potential. Furthermore, relocation of the 5-amino group in the dimethylamino derivative (5′-NH_2_ 4′-NMe_2_) resulted in a decrease in the activity. The tested 5-aminoflavones (5′-NH_2_ and 4′-NH_2_; 5′-NH_2_ and 4′-NHMe; 5′-NH_2_ and 4′-NHEt) demonstrated antitumor activity that was highly selective for the ER-positive breast cancer cell line, showing no effects against ER-negative human cancer cell lines, like HeLa S_3_, WiD, and MDA-MB-553.

**Figure 3:**
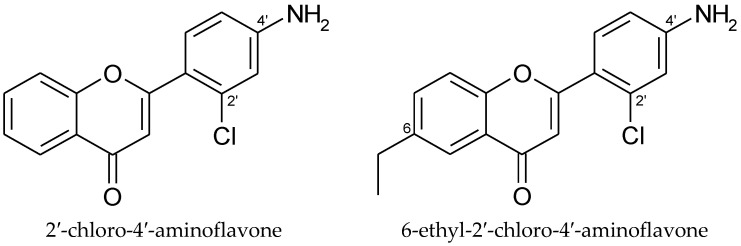
Chemical structure examples of 2′-chloro-4′-aminoflavones.

Jin et al. [34] obtained a series of 2′-chloro-4′-aminoflavones and tested them for their antiproliferative activity against hepatocarcinoma cells (HepG2), breast adenocarcinoma cells (MCF-7), and human chronic myelogenous leukemia cells (K562). The introduction of alkyl groups into the A-ring, such as methyl and ethyl ones as weak electron-donating groups, provided better antitumor activities. The best results were noted for 6-ethyl-2′-chloro-4′-aminoflavone (Figure 3) (IC_50_ = 1.8 µM), which was parallel to colchicine, which served as the standard. Furthermore, it was noted that this compound had very low toxicity against normal human liver cells QSG7701 and HL7702. At a concentration of 100 µM, the inhibition rates were 96.4% and 99.2%, respectively. Further study demonstrated that these compounds induced apoptosis in HepG2 cells. Cell-cycle arrest at the G2/M phase was observed in the range from 16 to 31% when increasing the concentration from 5 to 50 µM. All of the compounds had no effect on breast adenocarcinoma cells MCF-7 (with IC_50_ > 50 mM).

**Figure 4:**
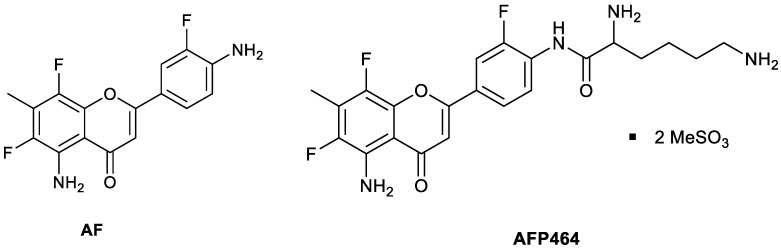
Chemical structure of AF and AFP464.

Some of the aminoflavones mentioned below have recently undergone evaluation in clinical trials. Brantley et al. [35] provided a rationale for the continued development of AFP464 (Figure 4), which is a precursor of aminoflavone, as an agent to enhance the therapeutic management of breast cancer via dysregulation of the aryl hydrocarbon receptor (AhR) signaling pathway.

AFP464 displays cytotoxicity and promotes AhR translocation from the cytosol to the nucleus in LM05 cells. It was found that AFP464 not only reduces bulk tumor cells, similarly to other P450 prodrugs, but also appears to target cells with stem-cell-like properties, at least in part by abolishing α6-integrin expression, which promotes metastasis.

Aminoflavone (AF), the active component of a novel anticancer prodrug (AFP464) in phase I clinical trials, is a ligand of the aryl hydrocarbon receptor (AhR). AhR dimerizes with HIF-1β/AhR, which is shared with HIF-1α, a transcription factor critical for the response of cells to oxygen deprivation. The response of mammalian cells to hypoxia is mediated, at least in part, by a family of transcription factors known as hypoxia-inducible factors (HIF). According to Terzuoli et al. [36], the inhibition of HIF-1α by the aminoflavone AFP464 was independent of a functional AhR pathway. Moreover, this compound was also inactive in MDA-MB-231 cells, yet inhibited HIF-1α in MDA-MB-231 cells transfected with the SULT1A1 gene.

**Figure 5:**
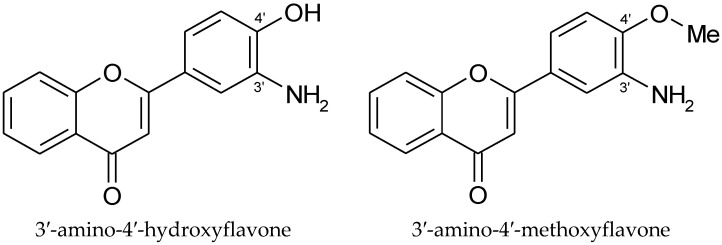
Structure of the 3′-aminoflavone derivatives.

Flavone itself and some aminoflavones have also been reported as inhibitors of protein tyrosine kinase in vitro. Król et al. [37] tested flavone (2-phenylbenzopyran-4-one) and amino-substituted flavones in vitro for the production of nitrite, a chemical product of nitric oxide, in model murine activated peritoneal macrophages. Among the tested compounds, 3′-amino-4′-hydroxyflavone (Figure 5) was the most potent inhibitor of nitrite production. 3′-Amino-4′-methoxyflavone demonstrated the most potent inhibition of the EGF receptor tyrosine kinase’s activity derived from A431 cells (42% inhibition at 50 µM).

**Figure 6:**
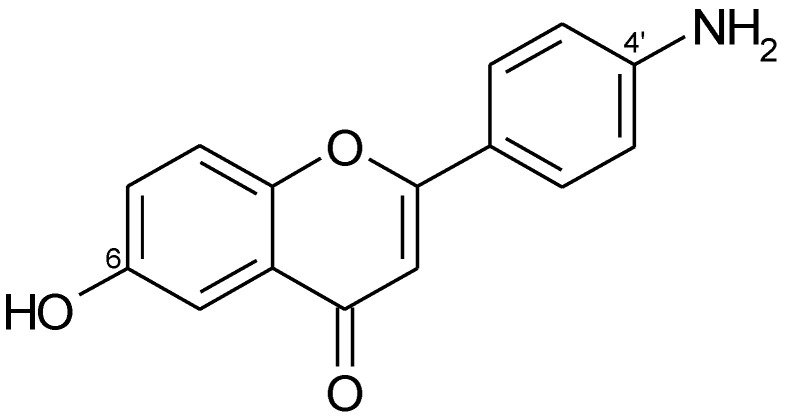
Structure of the 4′-amino-6-hydroxyflavone.

Moreover, Cushman et al. [38] reported a series of 4′-aminoflavones which inhibited the activity of the protein tyrosine kinase. The most potent compound was 4′-amino-6-hydroxyflavone (Figure 6), which inhibited this activity with an IC_50_ = 1.2 µM.

**Figure 7:**
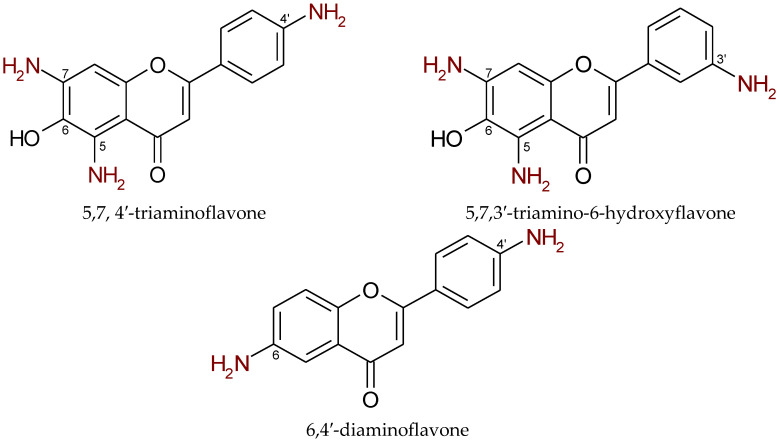
Chemical structures of aminoflavones with anti-inflammatory action.

These and further results [18] suggest that aminoflavones may modulate inflammatory and immune responses by controlling the production of nitric oxide. The amino group therefore evidently plays a critical role in allowing recognition, because none of the tested nitroflavones inhibited the activity of the enzymes p56^lck^, EGF_r_, and p60^v-src^ (Figure 7).

The results emphasize the importance of the 5,7-diamino-6-hydroxy substitution pattern, because the most active compounds in the tested series against p56^lck^ (5,7, 4′-triaminoflavone, 6-hydroxyflavone and 5,7,3′-triamino-6-hydroxyflavone) showed an IC_50_ equal to 18 µM and 48 µM, respectively. When tested against EGFr, the compounds 6,4′-diaminoflavone and 5,7,3′-triamino-6-hydroxyflavone showed an IC_50_ of 8.7 and 7.8 µM, respectively. These compounds were also the most active against p60^v-src^ (IC_50_ between 28.8 and 38.4 µM).

**Figure 8:**
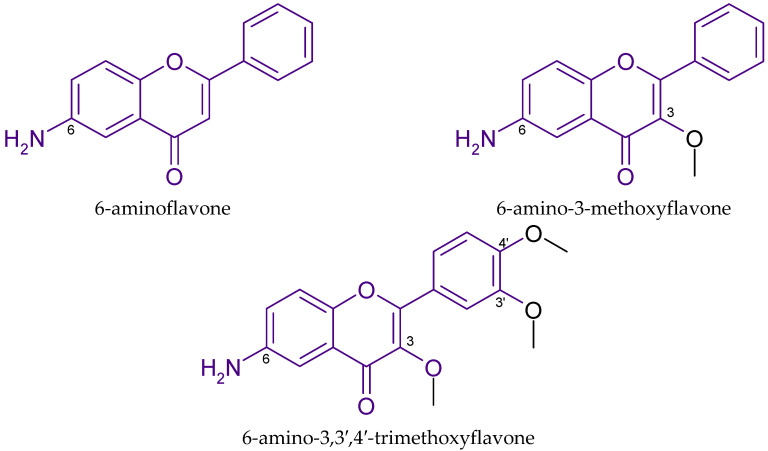

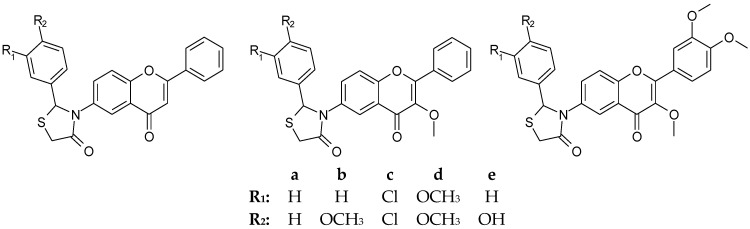
6-Aminoflavonoids and their thiazolidinone analogs with antitumor effects.

So as to enhance the antitumor activity, heterocyclic analogs have also been synthesized by combining the amino flavonoid ring and the ring of thiazolidine compounds. To study the aminoflavone derivatives, several compounds differing in the number of methoxy groups, such as 6-aminoflavone, 6-amino-3-methoxyflavone, and 6-amino-3,3′,4′-trimethoxyflavone, were used (Figure 8) [39].

The compounds were tested for their antitumor activity in vitro and in vivo. The research confirmed the cytotoxic potential of new analogs to the human breast adenocarcinoma MDA-MB-435 and HeLa cells. Interestingly, the presence of methoxy groups increased the cytotoxic potential against the cancerous cells without being toxic to healthy ones. As expected, the synthesized compounds increased the lifespan of mice with cancer.

According to recent studies [40], chrysin modified at the 7-position via regioselective alkylation to 7-aminochrysin derivatives showed better anticancer effects than the substrate. Menawhile, the transformation of *N*-phenylacetamides to the corresponding diphenylamine-type compounds decreased the anticancer activity. The promising results were obtained for the product of the reaction of chrysin with 2-chloro-*N*-(3,5-dimethoxyphenyl) acetamide, which showed the highest cytotoxic activity against the MCF-7 cell line of breast cancer (GI_50_ = 30 nM) and the HCT-15 cell line of colon cancer (GI_50_ = 60 nM).

**Figure 9:**
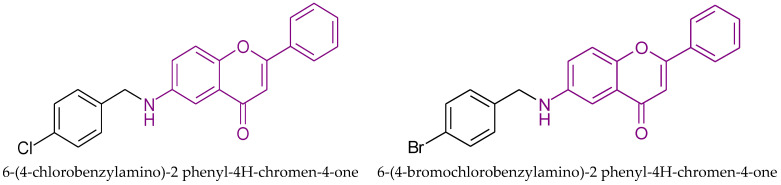

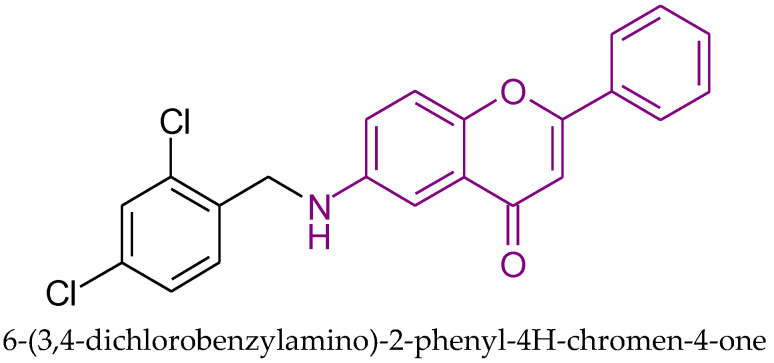
6-Benzylaminoflavones.

Thorat et al. [41] synthesized a series of novel *N*-benzyl derivatives of 6-aminoflavone (Figure 9) and evaluated them for their anticancer activity (against the MCF-7 and A-549 cell lines) and for their topoisomerase II enzyme inhibition activity. Compounds with 4-chloro and 4-bromo substitution on the beznylamino group 6-(4-chlorobenzylamino)-2-phenyl-4H-chromen-4-one and 6-(4-bromobenzylamino)-2-phenyl-4H-chromen-4-one) were found to be the most potent anticancer agents against MCF-7 cells with an IC_50_ = 9.35 µM and 9.58 µM, respectively. These compounds were also found to exhibit robust inhibition of enzyme topoisomerase II with an IC_50_ of 12.11 and 12.79 μM, respectively. Meanwhile, the compound with a 3,4-dichloro substituent on the benzylamino group (6-(3,4-dichlorobenzylamino)-2-phenyl-4H-chromen-4-one) showed good inhibition (46.48%) of the human lung cancer cell line A-549.

**Figure 10:**
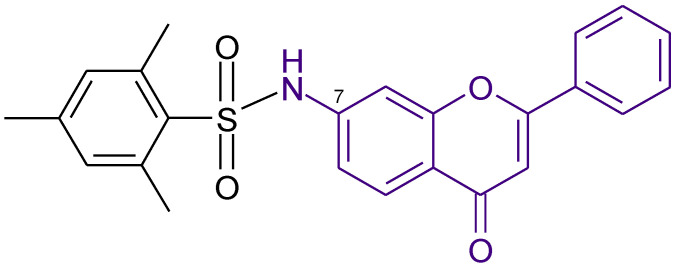
7-Aminoflavone’s sulphonamide structure.

Another research team came to a similar conclusion. Shelke et al. [42] synthesized a series of new sulphonamide analogs of 6- and 7-aminoflavones (Figure 10) and tested them against cancer cell lines (HepG-2, A-549, and Caco-2). Some of the obtained compounds demonstrated excellent antiproliferative activity (IC_50_ = 0.98 µM), which was comparable to the standard drug adriamycin (IC_50_ = 0.94 µM).

The authors documented that these compounds exhibited anticancer activity via the inhibition of topoisomerase II, thus inducing DNA damage in cancer cells (Table 2).

More recently, 6-aminoflavone was proposed by Flores-Flores et al. [43] as a potential agent for the treatment of asthma and related respiratory diseases. It exhibits a significant relaxant activity via calcium channel blockade, and a molecular docking study revealed that it binds to the L-type calcium channel. The same compound was examined by Ahmad et al. [44] as a potent drug that reduces D-galactose-induced oxidative stress, which reduces neuroinflammation. These authors declared that 6-amimoflavone can be viable therapeutic agent for addressing memory loss.

## 4. Aminoflavone Complexes

**Figure 11:**
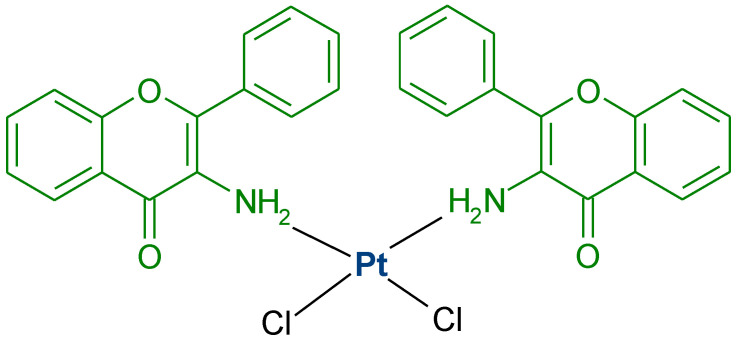
Complexes of 3-aminoflavones with cisplatin.

The medicinal properties of naturally occurring flavonoids have been well known for many years. However, the discovery that their complexes with metal ions are more effective than flavonoids themselves has changed the course of drug research. Flavonoids act as chelating agents due to the presence of carbonyl oxygen and the substituents with free electron pairs, e.g., hydroxyl or amine groups [45,46]. These results have shown that these complexes can be successfully used in the therapy of a number of diseases, such as diabetes, bacterial infections, and cancer. Their role in the treatment of neurodegenerative diseases, such like Huntington’s disease, is also mentioned. Such complexes can also affect the iron balance in living organisms, which is important in the treatment of such diseases as Friedreich’s or Thalassa’s ataxia [47]. There are also known complexes of aminoflavones with cisplatin (Figure 11), a commonly used anticancer drug. In these complexes, 3-aminoflavone is used as the ligand instead of ammonia. The obtained derivatives are characterized by a high antitumor activity, e.g., in relation to ovarian tumor cells (CAOV 3, OVCAR 3) and lung cancer (A549).

**Scheme 4:**
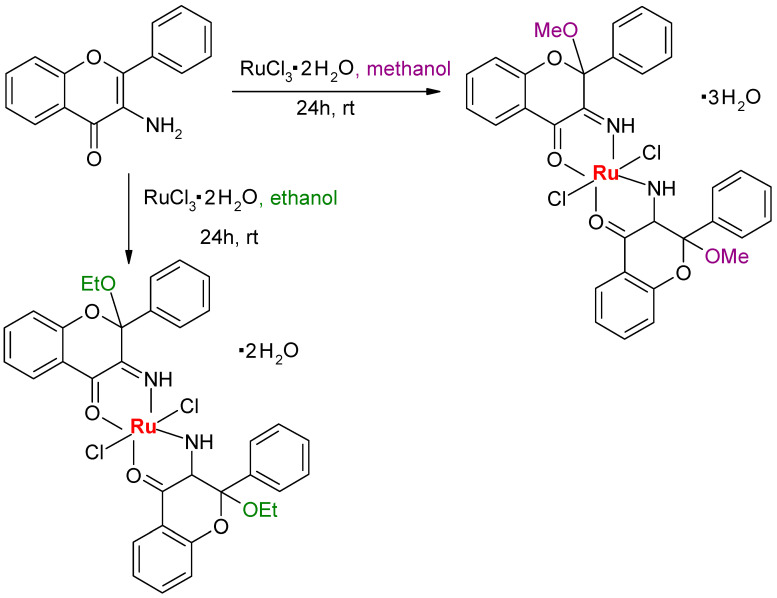
Metal complexes of aminoflavonoids.

Antitumor activity was also reported for two new flavanone ruthenium (II) complexes, formed in the reaction of ruthenium (II) chloride with 3-aminoflavone dissolved in aliphatic alcohol. 3-Aminoflavone was first oxidized and then solvolyzed to give 3-imino-2-alkoxyflavanone, which served as the ligand for the ruthenium ion (II) (Scheme 4) [48].

The pharmacological activities of the resulting complexes were evaluated against human bladder cancer cells. This study showed that the obtained compounds are highly cytotoxic against cancer cells. At the same time, the in vitro study proved the low toxicity of the synthesized complexes to healthy human lymphocytes.

Orzechowska et al. [49] analyzed the effect of the *trans*-platinum(II) complex of 3-aminoflavone on the viability and mortality of the OVCAR 3 and CAOV 3 ovarian cancer cells and on the expression of the selected genes involved in the process of apoptosis. It was noted that an increased concentration of the new metal–flavonoid complex resulted in a decrease in viability and an increase in mortality of the ovarian cancer cells. Increased Bax gene expression and decreased expression of Bcl2 and BIRC5 genes in the ovarian cancer cells were observed after the treatment with trans-Pt(3-af)₂Cl₂.

## 5. Aminochalcones

**Figure 12:**
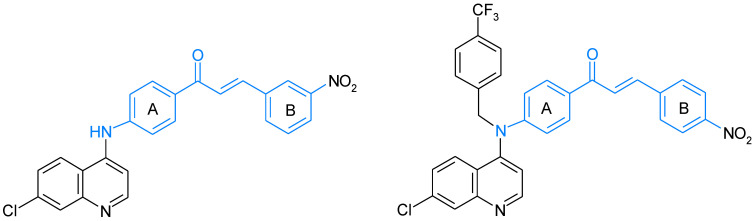
Hybrid derivatives incorporating arylnitro and aminochalcone moieties with antiprotozoal effects.

Chalcones, intermediates in flavonoid biosynthesis, play an important role as natural pigments found in many plants. Having a simple typical skeleton bearing two phenyls (rings A and B) spaced by a *trans*-enone bridge (1,3-diaryl-2-propen-1-one skeleton) (Figure 12), these compounds are important scaffolds in medicinal chemistry [50].

**Scheme 5:**
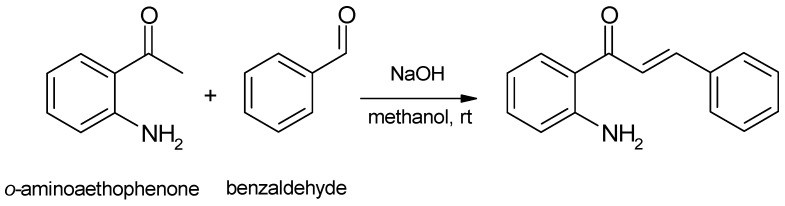
One-pot synthesis of aminochalcone involving Claisen–Schmidt condensation.

The presence of an *α*,*β*-unsaturated keto functionality (2-propen-2-one chain) seems to be responsible for the medicinal properties of the compounds. Depending on the substitution pattern in the phenolic rings, they differ in their biological properties, which mainly comprise anti-inflammatory, neurodegenerative, antifungal, and antibacterial activity [51,52,53,54]. Aminochalcones do not occur naturally. They may be synthesized in a one-pot synthesis involving Claisen–Schmidt condensation (Scheme 5), followed by the chemoselective hydrogenation of the nitro group of the nitrochalcone [55].

Potency against cancer cells has been reported for chalcones substituted with amino groups.

A range of chalcones with various electron-withdrawing and electron-donating substituents have been evaluated for their anticancer activity. As a result, a potent and selective cytotoxic effect of 2′-aminochalcone bearing an unsubstituted B ring was observed. Several recent studies have focused on the evaluation of the biological activity of chalcones as potential cytotoxic agents against canine cancer cells. These revealed a potent activity of unsubstituted chalcone and 4′-methoxychalcone against the canine macrophage tumor cell line, which exhibited time-dependent and concentration-dependent cytotoxicity.

**Figure 13:**
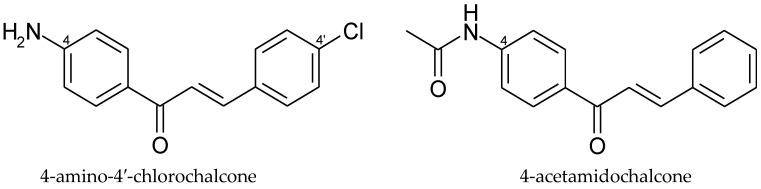
Selected chalcones tested against *Trichophyton* species.

Tristão et al. [56] reported a series of chalcone derivatives with a nitrogen atom to be antimicrobial agents. Their study showed that 4-aminochalcones exhibited antifungal effects. The aminochalcones were more toxic than the acetamidochalcones, while the nitrochalcones did not have any toxic effect. The results suggested that the antimicrobial activity to a large extent was governed by the groups attached to the aromatic B ring (furanyl, thiophenyl, chloro and methoxy ones). 4-Amino-4′-chlorochalcone (Figure 13) proved to be an excellent growth inhibitor for the cultured *Trichophyton mentagrophytes* ATCC 9973 and *Trichophyton rubrum* C137 (MIC = 20 µg/mL and 60 µg/mL, respectively).

Meanwhile, 4-aminochalcones with either furanyl or thiophenyl group at the C-4 position in ring B were found to inhibit the growth of the pathogenic fungus *Aspergillus niger* ATCC 9092. 4-Acetamidochalcone showed measurable levels of minimum inhibitory concentration of 20 µg/mL against *Microsporum canis* C112, *Trichophyton mentagrophytes* ATCC 9972, and *Trichophyton rubrum* C137.

**Figure 14:**
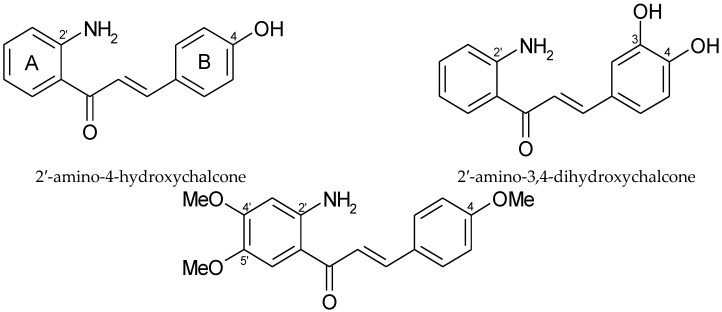
Selected 2′-aminochalcones with antioxidant effects.

Sulpizio et al. [57] tested 2′-amino-4-hydroxychalcone and its analog 2′-amino-3,4-dihydroxychalcone (Figure 14) in order to assess their antioxidant activity using a DPPH radical assay.

The results indicated that the aminochalcone carrying two hydroxyl functionalities in the adjacent meta and para positions in ring B exhibited stronger antioxidant activity than other derivatives. The aminochalcone with two hydroxy groups in ring B showed an IC_50_ value (the compound concentration necessary to decrease the initial amount of DPPH by 50%) of 4.9 ± 1 µM. The results indicated that the presence of the two hydroxyl groups in ring B is important for the antioxidant activity of the aminochalcones. Additionally, it was evidenced that a significant inhibition of DPPH occurs only in the case of the two hydroxyl groups in meta and para positions in ring B, which suggests that the amino group is not involved in the antioxidant activity and that the hydrogen atom transfer comes from the phenolic group rather than from the ketoenolic moiety. The analogs having only one hydroxyl group or the methoxyl substituent in ring B proved to be less effective. The differences in biological activity of 2′-aminochalcones may arise from the formation of a side product via cyclization between the amino group in ring A and the aromatic B ring.

**Figure 15:**
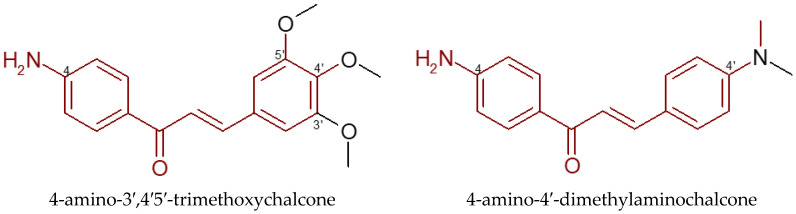
Chemical structures of 4-aminochalcones.

Prasad et al. [58] tested the antioxidant potential of para-substituted aminochalcones. Based on the results of this study, it can be inferred that substitution of the aromatic ring B with electron-donating groups at the ortho and/or para positions will increase the antioxidant activity of 4′-aminochalcones. The study results unequivocally show that 4-amino-3′,4′5′-trimethoxychalcone and 4-amino-4′-dimethylaminochalcone (Figure 15) have significant antioxidant activities, proven both in reactive oxygen species assays and in inhibiting the lipid peroxidation. These results suggest that the electron-releasing pharmacophores, such as the methoxy or dimethylamino groups, may be essential for the antioxidant activity.

Moreover, Apiraksattayakul et al. [53] recently determined that methoxy derivatives of 4-aminochalcone exhibit neuroprotective effects against H_2_O_2_-induced oxidative stress in human neuroblastoma SH-SY5Y cells and they have potential as neuroprotective agents.

The study on structure–activity relationships carried out by Liu et al. [59] revealed that the chalcone skeleton is the most preferable for the anticancer activity. The in vitro cytotoxic activity measured by the MTT assay indicated that chalcone derivatives had higher activity than the corresponding flavones, with all IC_50_ values being lower than 10 µg/mL against all tested human tumor cell lines (ESA-109, A-549, HL-60, and PC-3).

**Figure 16:**
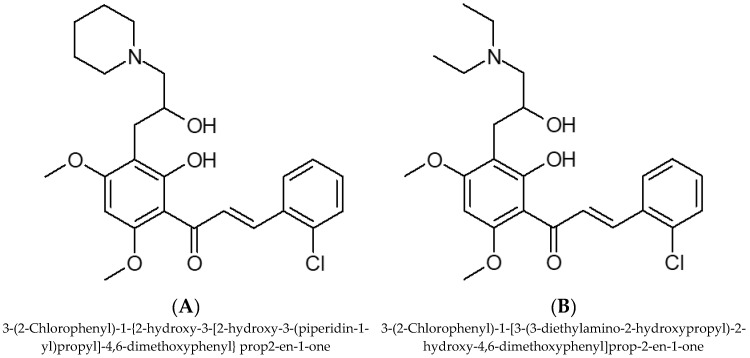
Chemical structures of selected chalcone derivatives.

Chalcone with a piperidinyl substituent at the C-3′ position (Figure 16A) was the most promising compound due to its high potency against the examined cancer cell lines (the IC_50_ values for ECA-109, A549, HL-60, and PC-3 cells were 1.3, 1.6, 2.6, and 2.5 μM, respectively). The introduction of open-chain aliphatic amino groups at C-3′, affording diethylamino, ethylmethylamino, and dimethylamino derivatives, led to the conclusion that it was a bulkier substituent that was favorable for the antiproliferative activity. The dietylamino derivative (Figure 16B) exhibited excellent antitumor activity against all the tested cell lines, with the IC_50_ ranging from 0.96 µM for HL-60 to 3.9 µM for PC-3 cells. Meanwhile, cyclization of the chalcones to the corresponding flavones and formation of the B ring led to a reduction in the cytotoxic activity.

Zeraik et al. [60] studied 4-aminochalcones substituted with fluorine, methyl, and hydroxyl groups in ring B and showed that three of these compounds were very interesting inhibitors of the chlorinating activity of myeloperoxidase. The compounds 4-aminochalcone, 4-amino-4′-fluorochalcone and 4-amino-4′-methylchalcone presented high oxidation potential, low scavenger capacity, and low inhibition of the respiratory burst of neutrophils. The activity of the tested chalcones was correlated with the lipophilicity of the substituents in ring B. The absence of a C-4′ amino group or its replacement with a hydroxyl one blocked the inhibitory effect. Biological activity screening of 2- and 4-aminochalcones showed the inhibitory activity of the compounds against canine malignant histiocytic cells (DH82). Aminochalcones with a hydrophobic substituent in ring B proved to be potent antiproliferative agents. A comparison of the unsubstituted *trans*-chalcone with 4-aminochalcone led to the conclusion that the amino group improved cytotoxicity against DH82 cells (inhibition of the cell growth by 27 and 59%, respectively, was noted). The presence of halogens and the methyl group at the para-position of the B ring also improved the activity. Replacement of the substituent at C-4′ with the strong electron-donating methoxy group resulted in a decrease in the cytotoxicity compared to 4-aminochalcone [27].

The anti-inflammatory and antioxidant properties of 2-aminochalcone, 3-aminochalcone, and 4-aminochalcone and their derivatives were examined by Iqbal et al. [61]. The ranking of activity for regio isomeric aminochalcones was determined as follows: orto > meta > para. It was observed that only the trifluoro-methyl analog showed better antioxidant activity than the unsubstituted chalcone. Good antioxidant potency was also observed for two aminochalcones (with 3-NH_2_ and 4-NH_2_ group) and for 4-cyanochalcone.

Aminochalcones obtained by Trein et al. [62] were evaluated for their activity against the parasitic protozoan *Trichomonas vaginalis*, which is the most common non-viral sexually transmitted disease worldwide. Among them, 3′-aminochalcone exerted the most potent effect and showed a high cytotoxicity against human vaginal cells. On the other hand, 3′-aminochalcone did not exhibit toxicity against *Galleria mellonella* larvae at a dose of up to 50 mg/kg over 120 h. Similarly, it had no hemolytic effect on human erythrocytes. When the erythrocytes were incubated with 3′-aminochalcone at a concentration of 100 µM, the result was not significantly different from the control. Trophozoites of *T. vaginalis* treated with this compound did not present significant reactive oxygen species (ROS) accumulation, but induced a significantly higher ROS accumulation in human neutrophils after co-incubation.

Xiao et al. [63] prepared a series of 4′-aminochalcones–revastimine hybrids and evaluated them as potential agents for the treatment of Alzheimer’s disease. They were tested for their cholinesterase inhibitory activity, antioxidant and metal-chelating effects, inhibitory effects on A*β* aggregation, and their ability to cross the blood–brain barrier (BBB). The results indicated that a compound containing one head group of dimethylamine at the 4′-position and a cyclic amine group at the C-4 position had the best inhibitory potency on acetylcholinesterase (IC_50_ = 4.91 µM) and significant antioxidant activity with a value 2.83-fold greater than that of Trolox. This molecule could also cross the BBB in vitro, possessed the ability to act as a metal chelator, and inhibited self-induced A*β*_1–42_ aggregation and Cu^+2^-induced A*β*_1–42_ aggregation.

**Figure 17:**
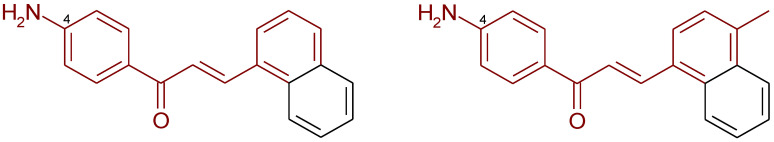
4-Aminochalcones with naphthyl moieties.

The results obtained by Seba et al. [64] indicated that 4′-aminochalcones with the naphthyl moieties 4-amino-4′-naphthylchalcone and 4-amino-4′-methylnaphtylchalcone (Figure 17) have potential anti-metastatic activity mediated by p53, which may be exploited for the treatment of osteosarcoma—the most recurrent malignant bone tumor, with high metastatic potential. It was confirmed that these compounds suppressed the migration of U2OS (p53 wt) cells and that of a SAOS-2 (p53 null) cell line expressing p53.

**Figure 18:**
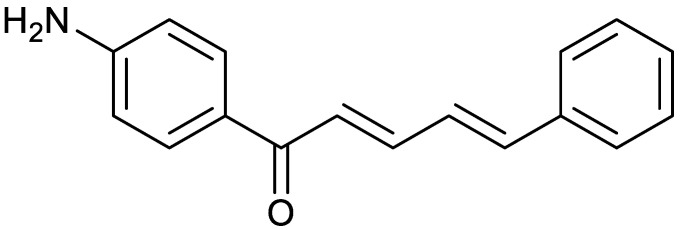
4-aminochalcone with an elongated aliphatic unsaturated chain.

Cavalcante et al. [65] obtained 4-aminochalcone and its derivative with an elongated aliphatic unsaturated chain (Figure 18) that presented trypanocidal effects, causing membrane damage and oxidative stress. These authors stated that the enlargement of the aliphatic unsaturated chain enhances the stability and lipophilicity of p-aminochalcones without affecting the trypanocidal effect. Both molecules induced membrane damage, oxidative stress, and mitochondrial dysfunction, leading to parasitic cell death.

Prasad et al. [66] synthesized a series of 4-aminochalcones via the Claisen–Schmidt condensation of 4-aminoacetophenone with various substituted aromatic aldehydes and tested them for their antimicrobial and anti-inflammatory activity. The highest antibacterial activity was noted for the compounds with chlorine at C-4′, bromine at C-3′, methoxy groups at C-3′ and C-4′, and also methoxy groups at C-3′, C-4′, and C-5′ in the aromatic ring B (the zone of inhibition was between 17 and 25 mm). All of the synthesized compounds exhibited moderate to considerable antifungal activity when compared to fluconazole, which was used as the reference standard. Some of them were found to also possess significant anti-inflammatory activity, comparable to standard drugs, such as aceclofenac.

However, some studies have revealed that we need to be careful when suggesting these compounds for therapeutic use. According to the toxicological study performed by Mariño et al. [67], all tested 4’-aminochalcones caused lower leucocyte growth compared to the negative control, as well as higher micronucleus generation in the mutagenic test (at 80 µM) than H_2_O_2_. In this study, the mutagenic and genotoxic effects were determined, which proved to be concentration-dependent.

Aminochalcones can also serve as the basis for obtaining other derivatives with a nitrogen atom, characterized by interesting biological properties. Patel and Dholakiya [68] synthesized new 4′-aminochalcone-based dibromomaleimides with antituberculosis and anti-candida activity.

Although there is some information in the literature on the biological activities of aminochalcone derivatives, additional studies on their safety are essential. Mariño et al. [67] evaluated the toxicity of 4′-aminochalcones in vitro using the cellular proliferation and viability assays, the micronucleus test, and the comet assay measuring DNA damage. Their results showed that the tested compounds have mutagenic and genotoxic effects, which are concentration-dependent.

## 6. Conclusions

The goal of the most recent research studies is to design new compounds that may be used in medicine as new pro-health pharmaceuticals, with reduced side effects. The increasing level of drug resistance among patients highlights the urgent necessity of new therapeutic options. Flavonoids containing a nitrogen atom, which constitute a large group of synthetic compounds, may be a promising source of new drugs and health-promoting active substances in the future. The aminoflavone prodrug AFP464 has been evaluated in clinical trials for the treatment of solid tumors, and its ability to suppress tumor-initiating cell (TIC) growth has also been suggested [69]. It not only reduces bulk tumor but also appears to target cells with stem-cell-like properties. On the basis of a recent critical review [70] regarding flavonoid drugs, it is known that nineteen flavonoid-based drugs have been approved and are available as medical prescriptions, and 36 candidates are undergoing or suspended in different clinical phases. Among them, three are undergoing different phases of pharmacological studies or are clinical candidates suspended in different phases of pharmacological tests.

Our summary showed that the synthetic or semi-synthetic modification of compounds with either a flavone or chalcone moiety can change their biological activities. Additionally, good cytotoxic and antioxidant properties of aminoflavonoids arise from their ability to form complexes via the chelation of metal ions. We hope that this review will facilitate the development of more potent active agents based on the scaffold of aminoflavonoids in the future. It is also important to continue the investigation of the mechanisms of their actions.

## Figures and Tables

**Table 1:** Antiproliferative activity of 5-aminoflavone’s derivatives against MCF-7 cells.

	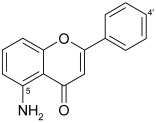 5-aminoflavone (5-AF)	
	IC_50_ [µM]	
	Estradiol (10^−4^ µM)	
	**−**	**+**
5-AF	>100	13
4′-OH	>100	1.5
4′-OMe	>100	12
4′-Br	9.5	1.0
4′-CN	94	>100
4′-COOH	>10	>10
4′-NH_2_	0.0098	0.0072
4′-NHAc	18	13
4′-NHMe	0.0054	0.0023
4′-NHEt	0.0017	0.0013
4′-NHCH_2_Ph	4.3	4.1
4′-NMe_2_	0.0050	0.0040
4′-NEt_2_	0.010	0.0020

**Table 2:** Anticancer activity of aminoflavonoids.

Aminoflavone Derivatives with Anticancer Effects			
Chemical Structure	Cell Line	IC_50_ (μM)	Reference
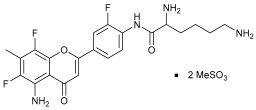	Human renal cancer cells		Luzzani et al. [29] Brantley et al. [36]
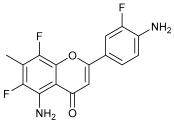	Human breast cancer cell lines		Brinkman et al. [31]
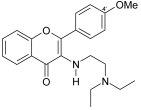	Murine L1210 leukemia cells	22	Dauzonne et al. [33]
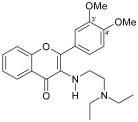	Murine L1210 leukemia cells	10	Dauzonne et al. [33]
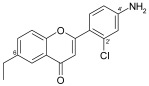	Hepatocarcinoma cells (HepG2), breast adenocarcinoma cells (MCF-7), and human chronic myelogenous leukemia cells (K562)	1.8	Jin et al. [35]
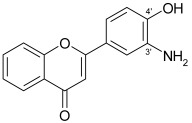	Inhibitors of protein tyrosine kinase		Król et al. [38]
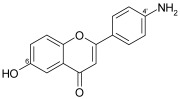	Inhibitors of protein tyrosine kinase	1.2	Cushman et al. [39]
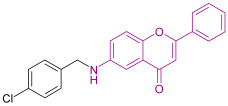	Anticancer activity to MCF-7 cells	9.35	Thorat et al. [41]
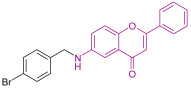	Anticancer activity to MCF-7 cells	9.58	Thorat et al. [41]
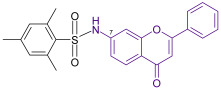	Anticancer to HepG-2, A-549, and Caco-2cell lines	0.98	Shelke et al. [42]

## Data Availability

Data are contained within this article.
